# Self-reported quality of life and self-esteem in sad and anxious school children

**DOI:** 10.1186/s40359-016-0153-0

**Published:** 2016-09-13

**Authors:** Kristin D. Martinsen, Simon-Peter Neumer, Solveig Holen, Trine Waaktaar, Anne Mari Sund, Philip C. Kendall

**Affiliations:** 1 Centre for Child and Adolescent Mental Health, Gullhaugveien 1-3, 0484 Oslo, Norway; 2 Department of Psychology, University of Oslo, Forskningsveien 3A, 0373 Oslo, Norway; 3 NTNU, Regionalt kunnskapssenter for barn og unge (RKBU), Klostergata 46, 7030 Trondheim/St. Olav’s Hospital, Trondheim, Norway; 4 Temple University, 1701 North 13th Street, Weiss Hall, Philadelphia, PA USA

**Keywords:** Quality of life, Self-esteem, Anxiety, Depression, Children at risk, Prevention

## Abstract

**Background:**

Anxiety and depressive symptoms are common in childhood, however problems in need of intervention may not be identified. Children at risk for developing more severe problems can be identified based on elevated symptom levels. Quality of life and self-esteem are important functional domains and may provide additional valuable information.

**Methods:**

Schoolchildren (*n* = 915), aged 9–13, who considered themselves to be more anxious or sad than their peers, completed self-reports of anxiety (Multidimensional Anxiety Scale for children (MASC-C), depression (The Short Mood and Feelings Questionnaire; SMFQ), quality of life (Kinder Lebensqualität Fragebogen; KINDL) and self-esteem (Beck self-concept inventory for youth (BSCI-Y) at baseline of a randomized controlled indicative study. Using multivariate analyses, we examined the relationships between internalizing symptoms, quality of life and self-esteem in three at-risk symptom groups. We also examined gender and age differences.

**Results:**

52.1 % of the screened children scored above the defined at-risk level reporting elevated symptoms of either Anxiety and Depression (Combined group) (26.6 %), Depression only (15.4 %) or Anxiety only (10.2 %). One-way ANOVA analysis showed significant mean differences between the symptom groups on self-reported quality of life and self-esteem. Regression analysis predicting quality of life and self-esteem showed that in the Depression only group and the Combined group, symptom levels were significantly associated with lower self-reported scores on both functional domains. In the Combined group, older children reported lower quality of life and self-esteem than younger children. Internalizing symptoms explained more of the variance in quality of life than in self-esteem. Symptoms of depression explained more of the variance than anxious symptoms. Female gender was associated with higher levels of internalizing symptoms, but there was no gender difference in quality of life and self-esteem.

**Conclusion:**

Internalizing symptoms were associated with lower self-reported quality of life and self-esteem in children in the at-risk groups reporting depressive or depressive and anxious symptoms. A transdiagnostic approach targeting children with internalizing symptoms may be important as an early intervention to change a possible negative trajectory. Tailoring the strategies to the specific symptom pattern of the child will be important to improve self-esteem.

**Trial registration:**

Trial registration in Clinical trials: NCT02340637, June 12, 2014.

## Background

Internalizing disorders of anxiety and depression are common [1–4], often comorbid [5, 6] and have an impairing influence on children’s everyday lives and functioning [7–9]. Symptoms, even though not reaching a diagnostic level, may put the child at risk for later developing full disorders [10]. Thus, several studies have shown that self-reported depressive symptoms in children have a strong prognostic power to predict subsequent depressive disorders in youths [10–12]. Likewise, childhood anxiety symptoms are a risk factor for adolescent anxiety and depressive disorders [10, 12–15]. Elevated symptoms of anxiety or depression may also interfere with school functioning and academic achievement, and these associations seem to be bidirectional [16].

Prevalence rates of anxiety and depressive symptoms may vary with age. Although some studies suggest that the overall prevalence rates of fears and anxiety decrease from preadolescence into adulthood [17], other studies indicate that there are different developmental trajectories for specific anxiety symptoms, such as separation anxiety and social anxiety [3, 18]. There also seems to be distinct gender differences, with females reporting more fears than males [17, 19]. Anxiety is often found to precede depression [7] and children showing mixed symptomatology may thus have had the problems for a longer time than having anxiety only. Furthermore, depressive symptoms in girls age 14–15 seem to increase more rapidly than for boys at the same age [20–22]. Both anxiety and depression may be precursors for other difficulties [8, 23] and even not qualifying for a full disorder, such symptoms may reduce daily functioning [24].

It should however be kept in mind that anxious or sad feelings are also normal aspects of life. For intervention purposes it is therefore necessary to differentiate between children at risk for developing psychopathology from children showing normal variations of feelings. Not providing service leaves early symptoms unattended and thereby runs the risk that children suffering from internalizing symptoms miss receiving adequate early help [22, 25–28]. Collecting children’s self-report of anxious and depressive symptoms is one way of identifying children in need of preventive interventions.

For some children elevated levels of symptoms of anxiety and depression may over time be associated with functional impairment and lower levels of well-being [29–31]. Quality of life and self-esteem are among the domains that in combination with elevated levels of internalizing symptoms could imply higher problem severity and thus indicate an at-risk sub sample of children that may be in need of indicated preventive efforts [32].

According to Mattejat et al. [33], quality of life can be defined as “a subjective perception of well-being and satisfaction that can best be evaluated by the child according to his or her own experience within several life domains”. The concept thus emphasizes a child’s subjective satisfaction with his or her functioning in everyday life [34]. During the last decade, a number of studies have addressed quality of life in children and adolescents with mental health problems [32, 34, 35]. A general finding is that children with mental health problems report lower quality of life compared to healthy children as well as those with a physical disorder [36, 37]. In a clinically based Norwegian study with children aged 8–15, those with anxiety/depression reported lower quality of life than did the AD/HD group [35]. Bastiaansen and collegues [34] found that anxiety disorders had a negative impact on quality of life similar to children with externalizing disorders and mood disorders.

Does quality of life add incrementally to the identification of health service needs that are not detected by symptoms alone? The results of one study [29] indicated that children in outpatient services reported significantly lower quality of life than children in the community with the same level of emotional and behavioral problems. The investigators concluded that for children with equal levels of mental health problems, quality of life measurement would add important information about the total severity of the condition and hence the need for an intervention.

Self-esteem can be described as an individual’s global evaluation of his or her overall worth as a person [38]. While some have argued that self-esteem and depression can be joined under the construct of negative emotionality as they share a large proportion of variance [39], others emphasize factors to the contrary and have argued for the importance of distinguishing between the two constructs [40]. In cognitive vulnerability models of anxiety and depression, a negative self-view is considered a risk factor that may increase the likelihood for onset of disorders [41]. Adolescence is a sensitive time with many developmental challenges, and research suggests that self-esteem decreases during these years, especially for girls [42, 43].

According to van Tuijl and colleagues [41], research on adolescent and adult samples has consistently suggested lower self-esteem in individuals with higher levels of depression and anxiety symptoms, e.g. [41, 44]. Furthermore, findings from a meta-analysis supported that low self-esteem is predictive of symptoms of depression and anxiety [45]. There is less knowledge on younger children, but a study of Mexican-origin children found low self-esteem to be a prospective risk factor of depression for children aged 10–12 [46]. Steiger and colleagues [38] emphasize the malleability of self-esteem during the adolescent years. It may be important for preventive interventions to target low self-esteem either indirectly through the negative self-related thoughts consistent with the symptomatology of anxiety and/or depression or directly through working with improving self-esteem.

Examining how symptoms of anxiety and depression are related to the child’s functioning by assessing self-reported quality of life and self-esteem may thus improve our understanding of at-risk children. By focusing especially on symptomatic sub-groups, we can determine if there are differential relations between symptom level and quality of life and self-esteem for children with different combinations of problems. Maybe having some symptoms imply higher problem severity, pointing at the importance of intervening for specific subgroups. If self-esteem is affected, this could also point at the importance of focusing on self-esteem in indicated interventions.

The present study examined symptoms of anxiety and depression at baseline as reported by a self-selected sample of school children aged 9–12 years. Children were invited to participate in the pretest by having information about the study presented in class and in appropriate letters to children and parents. Children were screened for participation in the randomized controlled trial studying the effect of a targeted preventive intervention to reduce symptoms of anxiety and depression in children. Children exhibiting symptoms of anxiety, depression or both above a normative mean, were included in the RCT based on recommendations from relevant studies [47–49]. Analyses in the present paper were also based on the same at-risk sample.

The associations of internalizing symptoms with self-reported quality of life and self-esteem were evaluated, controlled for gender and grade level in the three different at-risk groups (i.e. children having symptoms of Anxiety only, Depression only or Combined (Anxiety and Depression). We hypothesized that there would be significant differences in means between the at-risk groups with regard to self-reported quality of life, self-esteem, anxiety, and depression and that having symptoms of both disorders would imply higher symptom levels and lower self-reported quality of life and self-esteem than having symptoms of either depression or anxiety alone. We also assumed that older children and girls would report lower quality of life and self-esteem across symptom groups.

Based on earlier findings it was furthermore hypothesized that elevated symptoms of either anxiety or depression would be negatively associated with quality of life and with self-esteem. In addition, having symptoms of both disorders concurrently was expected to have a stronger relationship with quality of life and self-esteem than having symptoms of either alone.

## Method

### Recruitment procedure

School children were recruited from primary schools after an open invitation to municipalities in urban and rural areas of Norway. The schools had agreed to participate in a randomized controlled study aiming to reduce the levels of anxious and/or depressive symptoms among school children through a new transdiagnostic group intervention based on cognitive behavioral therapy. Identification of children at risk for developing disorders requires a screening procedure. This procedure must be acceptable to the ethical board, the school administration, to parents and their children. Since screening entire age groups of children for symptoms is neither usual nor seen as acceptable in Norway, the children and their parents were informed about the study at school and in parent meetings. It was emphasized that the target group for the study were children who believed they were more anxious and sad than their peers and their parents. Children expressing interest and who had informed consent from their parents were then invited to screening. The child’s scoring of 1 SD or more on symptoms of anxiety, depression or both, was considered the inclusion criteria for further participation in the RCT condition. The mean scores and standard deviations for inclusion on self-reported symptoms was based on population studies using unselected samples [48, 49]. Only the screened children with scores above the cutoff at pretest (*n* = 477) were included in the present study. The sample on which the current study is based was thus recruited from a subgroup of the total population, and should therefore have more problems than the normal population of children in this age group. In indicated prevention, this is however a necessary recruitment procedure as we want to target children who have a certain level of specific problems.

### Participants

In participating primary schools (*n* = 30) a total of 4.315 children in 4th–6th grade (9–12 years of age) and their parents were invited. The number of children screened were *n* = 915, and the analysis representing baseline data are based on the at-risk samples (*n* = 477) scoring 1 SD or more on symptoms of anxiety, depression or both. For details of the RCT go to https://clinicaltrials.gov/ct2/show/NCT02340637, Trial registration: NCT02340637, June 12, 2014.

### Measures

Multidimensional Anxiety Scale for Children (MASC-C). Anxiety symptoms were measured by the MASC-C [50], a 39-item, child self-report, assessing anxiety in youth between 8 and 19 years. The measure has four subscales: Physical Symptoms, Social Anxiety, Separation Anxiety/Panic and Harm Avoidance. The response options are “0” for “never true about me”, “1” for “rarely true about me”, “2” for “sometimes true about me” and “3” for “often true about me”. The MASC-C has high retest reliability [51, 52], and good predictive and discriminative validity [53–55]. Elevated scores are significantly associated with meeting diagnostic criteria in a Norwegian sample [56]. In this study, the total anxiety score of the MASC-C was used to indicate symptom-level of anxiety [50]. The total anxiety score was also used as a dichotomized variable, indicating whether the child scored above the defined cutoff or not. Given the variation in mean scores between boys and girls in unselected samples, we used gender specific cutoffs for anxiety [49]. MASC-C girls; X = 46 (SD 15), 1 SD above mean; ≥ 61 points, MASC -C boys; X = 39 (SD 15), 1 SD above mean; ≥ 54 points. Internal consistency of the MASC-C in the present study was high with Cronbach’s Alpha 0.91.

Short Mood and Feelings Questionnaire (SMFQ). Depressive symptoms were assessed by the SMFQ [57], a brief 13-items scale assessing cognitive, affective and behavioral-related symptoms of depression in children 8 to 18 years. Statements are rated as being either “true” (2), “sometimes true” (1), or not true (0). In a study of 8–16 years-olds [57] the SMFQ discriminated clinically referred youth from unselected pediatric controls, and depressed youth from non-depressed youth. The measure has recently demonstrated Norwegian norms for 8 to 15 year olds, high retest reliability (*r* = 0.8) and good content validity [22, 58]. A full-scale sum score was created as the sum of all the individual values [57]. In addition, a dichotomized variable was used, indicating whether the child scored above the decided cutoff or not.

The literature suggests the same mean to be used for boys and girls for inclusion of depressive symptoms in this age-group [47, 48]. SMFQ cut-off: X = 3.8 (SD 3.6), 1 SD above mean; ≥ 7 points. Internal consistency of the SMFQ in the current study, Cronbach’s alpha, was 0.94.

Beck Youth Inventory-II (BSCI-Y). Self-esteem was assessed using a subscale of the BSCI-Y [59]. The BSCI-Y measures self-concept in children between 7 and 18 years using 20 items, and is considered useful for screening in schools [59]. The self-concept inventory measures the child’s perception of self, body image, competence and relation to others. Statements are rated on a four-point scale, “1” for “never”, 2” for “sometimes”, “3” for “often” and “4” for “always”. Gender differences have been found, and the scale is divided into three age groups with different norms [60]. The total sum score based on all items was used [59] The inventory has Norwegian norms and the reliability of the Norwegian version was high (Cronbach’s alpha in the 0.8–0.9 range). Cronbach’s alpha in the current study was 0.93.

KINDL (Kinder Lebensqualität Fragebogen) [61] http://www.kindl.org/. The KINDL was used to assess quality of life. The KINDL was developed for epidemiological use in children and adolescents aged 4–16 years. It consists of 24 items and measures physical and emotional wellbeing, self-esteem, and social functioning (family, friends and school) on a 1–5 scale where 1 indicates “never” and 5 indicates “all the time”. The KINDL questionnaire is analyzed by adding the item responses marked on each sub-scale, transforming the scores to standardized scores enables comparisons to be made with norm data [62]. A mean of 81.9, SD 9.07 is reported from a normative sample of school children (*n* = 846) [63].

In a study with children aged 8–16 years, a Norwegian version of the KINDL showed satisfactory internal consistency and retest reliability of the KINDL total quality of life scale [64]. Cronbach’s alpha in the current study was 0.89.

Associations between the measures were expected as they measure related constructs. To investigate this issue, the strength of the relationships between the constructs were calculated using Pearson’s correlation, see Table 1. All associations were significant at *p* < .001. The moderate degree of associations however indicated that they still measure different concepts. The relatively week correlation (*r* = .353) between the independent variables (anxiety and depression) indicated low risk of multi-collinearity in the regression analysis.

**Table 1:** Correlations between anxiety, depression, quality of life and self-esteem

	Anxiety (MASC-C)	Depression (SMFQ)	Quality of life (KINDL)
Depression (SMFQ)	,353^**^		
Quality of life (KINDL)	-,430^**^	-,635^**^	
Self-esteem (BSCI-Y)	-,282^**^	-,500^**^	,698^**^

Note: *N* = 477. *KINDL* Kinder Lebensqualität Fragebogen, *BSCI-Y* Beck youth inventory-II-self-concept scale, *MASC-C* the multidimensional anxiety scale for children – child version, *SMFQ* the SMFQ (The Short Mood and Feelings Questionnaire); ** *p* < .001

The children screened was categorized into 3 at-risk groups depending on their scores on symptoms of anxiety and depression: the Anxiety only group scored ≥1 SD above the normative mean on anxiety symptoms only, the Depression only group scored ≥1 SD above the normative mean on depressive symptoms only, and the Combined group scored ≥1 SD above the normative mean on both anxious and depressive symptoms.

### Statistics

One-way between groups analysis of variance (ANOVA) (the statistical package IBM SPSS; version 22) compared the overall as well as the contrast differences in mean scores on quality of life, self-esteem, anxiety and depression within the at-risk groups. Multiple regression analysis assessed the degree of relationship between anxiety and depression on quality of life and self-esteem, controlling for gender and grade-level within each symptom group.

## Results

All children screened were *n* = 915, of them 53.7 % (*n* = 491) were girls.

More than half (52.1 %, *n* = 477) of the full sample scored >1 SD above the cutoff on symptoms of anxiety, depression or both. There were more girls (*n* = 277, 58.1 %) than boys (*n* = 200, 41.9 %) in the at-risk sample. The largest at risk-group (*n* = 243, 26.6 %) were children reporting symptoms of both anxiety and depression (the Combined group), 15.4 % (*n* = 141) reported symptoms of Depression only and 10.2 % (*n* = 93) reported symptoms of Anxiety only, see Table 2.

**Table 2:** Gender and group differences in self-reported quality of life, self-esteem, anxiety and depression

		Anxiety only (1)			Depression only (2)			Anxiety and depression (3)			Hochberg GT2
		(N: boys =45; girls = 48)			(N: boys =60; girls = 81)			(N: boys =95; girls = 148)			Diff. bw groups
		M	SD	95 % CI	M	SD	95 % CI	M	SD	95 % CI	
Quality of life (KINDL)	Boys	73.4*	7.6	(71.1; 75.6)	65.1	11.4	(62.1; 68.0)	58.6**	11.8	(56.2; 61.0)	3<2<1***
	Girls	69.5	8.0	(67.1; 71.8)	63.0	9.5	(60.9; 65.1)	53.4	12.2	(51.4; 55.4)	
Self-esteem (BSCI-Y)	Boys	42.2	7.0	(40.1; 44.3)	37.8	9.5	(35.4; 40.3)	34.4*	10.1	(32.4; 36.5)	3<2<1***
	Girls	40.7	9.1	(38.0; 43.3)	35.4	7.9	(33.6; 37.1)	30.6	8.5	(29.2; 32.0)	
Anxiety (MASC-C)	Boys	61.8	7.0	(59.7; 63.9)	43.8	7.2	(42.0; 45.7)	66.7	9.9	(64.7; 68.7)	3>1>2***
	Girls	68.3**	7.6	(66.1; 70.5)	51.9**	7.1	(50.4; 53.5)	74.8**	9.7	(73.2; 76.3)	
Depression (SMFQ)	Boys	4.2	1.7	(3.7; 4.7)	9.4	3.6	(8.5; 10.3)	11.4	3.9	(10.7; 12.2)	3>2>1***
	Girls	3.7	1.8	(3.2; 4.2)	9.9	2.9	(9.3; 10.5)	13.4**	4.7	(12.6; 14.2)	

Note: *N* = 477. *KINDL* kinder Lebensqualität Fragebogen, *BSCI-Y* Beck youth inventory-II-self-concept scale, *MASC-C* the multidimensional anxiety scale for children – child version, *SMFQ* the SMFQ (The Short Mood and Feelings Questionnaire); *p < .05, **p < .001 for gender differences, *** Hochberg GT2 indicates only significant differences at *p* < .001

### Group and gender differences

One-way between groups ANOVAs were conducted to examine if there were significant overall differences in means between the at-risk groups with regard to self-reported quality of life, self-esteem, anxiety, and depression. Hochberg GT2 was used in the contrast analysis as the groups were of different sizes, and the differences between the groups are indicated in Table 2.

We found a significant overall difference in self-reported overall mean scores on quality of life in the groups F (474, 2) = 76.6, *p* < .001). Children reporting both anxiety and depression (the Combined group) reported significantly lower quality of life than children in the Depression only group did (M_Combined_ = 55.5 vs M_Depression only_ = 63.9, *p* < .001, GT2 = 8.4, *p* < .001). The children in the Depression only group reported significantly lower quality of life than the Anxiety only group (M_Depression only_ = 63.9, vs M_Anxiety only_ = 71.3, *p* < .001, GT2 = 7.5, *p* < .001.

There was also an overall significant difference in means between the groups with regard to self-reported self-esteem F (474, 2) = 38.6, *p* < .001). Children in the Depression Only group reported significant lower self-esteem compared to children in the Anxiety only group (M_Depression only_ = 36.4, vs M_Anxietyonly_ = 41.4, *p* < .001, GT2 = 4.9, *p* < .001) and between the Combined group and the Depression only group there was also a significant difference with the Combined group reporting lower self-esteem than the Depression only group (M_Combined_ = 32.1, vs M_Depression Only_ = 36.4, *p* < 001, GT2 = 4.3, *p* < .001).

In addition, we found a significant overall difference in mean symptom level of anxiety, F (474, 2) = 270.7, *p* < .001). Post hoc analyses of contrast effects indicated a significant difference between self-reported anxiety in the Combined group compared to the Anxiety only group (M_Combined_ = 71.6 vs M_Anxiety only_ = 65.2, *p* < .001, GT2 = −6.5, *p* < .001). The Depression only group also self-reported on anxiety symptoms, and as expected they reported significantly lower anxiety scores than the Anxiety only group (M_Depression only_ = 48.5 vs M_Anxiety only_ = 65.2, *p* < .001, GT2 = 16.7, *p* < .001). Self-reported mean scores on depression were also significantly different across the groups F (474, 2) = 184.5, *p* < .001). Scores in the Combined group was significantly higher than in the Depression only group ((M_Combined_ = 12.6 vs M_Depression only_ = 9.7, *p* < .001, GT2 = −2.9, *p* < .001). The Anxiety only group also reported on symptoms of depression, and their depression scores were significantly lower than in the Depression only group (M_Depression only_ = 12.6 vs M_Anxiety only_ = 3.9, *p* < .001, GT2 = −5.8, *p* < .001).

We found significant gender differences in mean scores in self-reported anxiety in the Anxiety only group F (1, 91) = 18.2, *p* < .001, and in the Combined group F (1, 241) = 39.4, *p* < .001 with girls reporting higher levels of anxiety than boys did. Also children in the Depression only group reported on anxiety symptoms and with gender differences F (1139) = 45.1, *p* < .001. In the Combined group, there was furthermore a significant effect of gender on depression (F (1, 241) = 11.2, *p* < .001, on Quality of life F (1241) = 10.8, *p* < .001 and on self-reported Self-esteem F (1241) = 10.5, *p* < .05 where girls reported higher levels of symptoms of depression, and lower quality of life and self-esteem. In the Depression only group there was no significant difference in scores between boys and girls with regard to quality of life, self-esteem and depression.

### Anxiety and depression in relation to quality of life and self-esteem

Separate multiple regression analyses were performed within the at-risk groups predicting quality of life and self-esteem apart using symptoms of anxiety and depression as dimensional independent variables. Analyses were controlled for gender and grade level.

#### Quality of life

Examining the sample in relation to quality of life, there was a statistical significant relation between self-reported symptoms of depression and quality of life in the Depression only group (β = −.45, *p* < .001) and in the Combined group with a standardized beta for symptoms of anxiety (β = −.32, *p* < .001), and for depression (β = −.36, *p* < .001), see Table 3 below. Symptoms of depression explained most of the variance, Part^2^ = 20.3. % in the Depression Only group, and Part^2^ = 9.7 % in the Combined group. In the Combined group, symptoms of anxiety explained 6.9 % of the variance in quality of life. Grade level was statistically significant in Combined group at the *p* < .05 level, where older children reported lower quality of life than younger children did. Gender was not significantly related to quality of life in any of the at-risk groups.

**Table 3:** Standard multiple regression analysis for at-risk groups on quality of life

	Quality of life								
	Anxiety only (*n* = 92)			Depression only (*n* = 141)			Anxiety & depression (*n* = 243)		
	Std β	95 % CI	Part^2^	Std β	95 % CI	Part^2^	Std β	95 % CI	Part^2^
Anxiety (MASC-C)	-.11	(.3, −.1)	.9 %				-.32**	(−.5, −.2)	6.9 %
Depression (SMFQ)				-.45**	(−1.9, −1.0)	20.3 %	-.36**	(−1.3, −.7)	9.7 %
Grade level	-.17	(−4.5, .4)	2.9 %	-.14	(−4.7, .1)	1.9 %	-.13*	(−4.4, −.5)	1.6 %
Gender	-.21	(−6.9, .2)	3.7 %	-.06	(−4.2, 1.9)	0.3 %	.01	(−3.0, 2.4)	-
	R^2^: .094			R^2^:.228			R^2^:.380		

Note: Quality of life: *KINDL* kinder Lebensqualität Fragebogen, *MASC-C* the multidimensional anxiety scale for children – child version, *SMFQ* the SMFQ (The Short Mood and Feelings Questionnaire) * *p* < .05, ** *p* < .001, Part^2^ = effect size

In the Anxiety only group, the relation between anxiety symptom level, grade level and quality of life was not significant.

There was a clear tendency that the Combined model explained most of the variance in Quality of life with 38 %, (F (238, 4) = 36.43, *p* < .001). The model for Depression only explained 23 % (F (137, 3) = 13.45, *p* < .001) and the Anxiety only model 9 %, (F (88, 3) = 3.03, *p* < .05).

#### Self-esteem

Examining symptom levels in the at risk groups with regard to self-esteem, there was a significant relation between symptoms of depression and self-esteem (β = −.34, *p* < .001) in the Depression only group and in the Combined group (β = −.34, *p* < .001), see Table 4. In both groups, symptoms of depression explained most of the variance: Part^2^ = 11.6 % and Part ^2^ = 8.4 % respectively. Grade level was only significant in the Combined group (β = −.15, *p* < .05) with the oldest children scoring lowest on self-esteem. Gender was not significantly related to self-esteem in any of the at-risk groups.

**Table 4:** Standard multiple regression analysis for at-risk groups on self-esteem

	Self-esteem								
	Anxiety only (*n* = 92)			Depression only (*n* = 141)			Anxiety & depression (*n* = 243)		
	Std β	95 % CI	Part^2^	Std β	95 % CI	Part^2^	Std β	95 % CI	Part^2^
Anxiety (MASC-C)	-.09	(−.1, .3)	.8 %				-.12	(−.2, .03)	0.8 %
Depression (SMFQ)				-.34**	(−1.4, −.5)	11.6 %	-.34**	(−.9, −.4)	8.4 %
Grade level	-.04	(−3.1, 2.1)	.2 %	-.13	(−3.9, .4)	1.7 %	-.15*	(−3.9, −.5)	2.2 %
Gender	-.14	(−5.9, 1.6)	1.5 %	-.12	(−4.6, .9)	1.1 %	-.09	(−4.1, .5)	0.8 %
	R^2^:.019			R^2^:.149			R^2^:.221		

Note: Self-esteem: *BSCI-Y* Beck youth inventory-II-self-concept scale, *MASC-C* the multidimensional anxiety scale for children – child version, *SMFQ* the SMFQ (The Short Mood and Feelings Questionnaire) * *p* < .05, ** *p* < .001, Part^2^ = effect size

There were no significant relations between the Anxiety only group and self-esteem.

The model explained 22.1 % of the variance in self-esteem in the Combined group (F (238, 4) = 16.9, *p* < .001) with symptoms of depression explaining most of the included independent variables (8.4 %). The model for the group Depression only explained 14.9 % of the variance in self-esteem (F (137, 3) = 7.9, *p* < .001), while there was a non-significant relation between anxiety and self-esteem F (88, 3) = .56, n.s.).

Additional analyses indicated no interaction effect between symptoms of anxiety and depression on quality of life or self-esteem.

## Discussion

The present study examined self-reported internalizing symptoms in a sample of children aged 9–12 years in relation to self-reported quality of life and self-esteem controlled for grade level and gender. The children were recruited as part of a randomized controlled intervention trial to be run in schools and baseline measures were used. We examined self-reported quality of life and self-esteem in relation to symptoms of anxiety and depression and discuss if such functional domains may give additional indications of how internalizing symptoms may have differential impact on different at-risk groups.

The children were considered to be at risk for developing further problems if they scored 1 SD or more above a normative mean based on unselected or population samples in other studies on symptoms of anxiety, depression or both. In this study most children reported symptoms of both anxiety and depression, while children reporting anxiety only was the smallest at-risk group. This is different from population based studies where anxiety problems usually are the most common emotional problem for this age group [2]. Our main finding regarding the associations with the two functional domains was that when progressing from the Anxiety only group, to the Depression only group and finally to the Combined group, there was a gradual increase in anxious and depressive symptoms and a decrease in quality of life and self-esteem. In multivariate analyses, significant associations were found between symptoms of depression as well as comorbid anxiety and depression and self-reported quality of life and self-esteem. This was according to our hypothesis. There was however a significant difference between the symptom groups Anxiety Only and Depression only where having depressive symptoms only indicated lower quality of life and self-esteem than having anxiety symptoms only. The symptoms level of the Anxiety only group was not significantly related to the two functional domains, despite that the mean score on quality of life was more than one SD below the normative sample of the measure [63]. When targeting both anxiety and depression in a transdiagnostic intervention, it may thus be important to emphasize therapeutic strategies targeting symptoms of depression especially both with regard to time spent and tailoring them to the characteristics of the individual child as these symptoms appear to be closely related to the severity experienced by the children.

Symptoms of depression explained most of the variance in relation to quality of life. Symptoms of depression like low mood, anhedonia and lowered energy might set a spiral of experiencing lower quality of life in many areas, both because depressive symptoms are associated with less activity and less joy, and because having a high level of depressive symptoms might distort the child’s conception of him- or herself, the context and the future. Only in the Combined group, older children reported significantly lower quality of life than younger children. We did not find a significant effect of gender in any of the other symptom groups which was contrary to our hypothesis, namely that girls would report lower quality of life than boys would. Other studies have reported gender differences in quality of life, with girls showing a greater decrease than boys did [64, 65], but this was not replicated in our study.

Symptoms of anxiety alone (the Anxiety only group) did not gain a significant relation to the children’s experience of life quality. This finding indicates that having more or less anxiety within the at-risk range is not necessarily associated with quality of life. Anxiety symptoms may affect more specific domains, and does not affect the quality of life to the same extent as when having depressive symptoms. The Anxiety only group was also the smallest at-risk group in the study, which may have influenced our results. Restriction of range could also be a factor to consider, however the variance in the symptom scores of Anxiety only group were acceptable compared to the Combined group. We thus found partial support for our hypothesis; having high levels of symptoms in both domains had a stronger negative impact on quality of life than having symptoms of anxiety alone.

According to Jozefiak and colleagues [32] the child’s self-reported quality of life may be an important indicator of the child’s well-being that can provide us with information regarding the child’s need for health services to a greater extent than symptom level alone. Based on the current sample, it appears that symptoms of depression alone, and symptoms of depression and anxiety together was significantly associated with the child’s quality of life and as such may indicate higher problem severity in need of intervention. When both symptom groups are targeted in a united preventive intervention, as less positive change may be expected in these groups compared to the Anxiety only group as implied by higher problem severity. As anxiety also is found to often precede depression in children [7], it may be hypothesized that children with a mixed symptom presentation have had their problems longer and hence is more difficult to change.

There were significant associations between symptoms of depression and self-esteem in the Depression only group and in the Combined group. This was according to our hypothesis. We found significant age differences as indicated by grade level only in the Combined group, older children reporting lower self-esteem than younger children did. There were no significant effects of gender. Children who reported symptoms of Depression only or both Anxiety and depression, reported self-esteem in the lower than average, to much lower range [59] which is an indication of severity.

Earlier studies have indicated that self-esteem decrease with increasing age and also that gender differences in self-esteem increase with increasing age [60]. Our findings with regard to gender may be explained by the fact that the children in this study were in the lower age range. Symptoms of depression explained most of the variance in both the Depression only and in the Combined group. Depressive symptoms thus seem to be related to a negative self-perception. This is not surprising as depression often is characterized by a negative global self-evaluation, which is also central in the concept of self-esteem of a person [38]. There is, however, support for viewing self-esteem and depression as separate constructs [40].

In the Anxiety only group, there were no significant relations between symptoms of anxiety, gender, grade level and self-esteem. This result may indicate that anxiety affects a narrower area of functioning and thus does not threaten the global self-evaluation of the child.

Our study suggests that symptoms of anxiety and/or depression are negatively related to the child’s self-perception. The importance of working to enhance a child’s self-evaluation in these at-risk groups especially is supported by existing research as low self-esteem is a risk factor for developing symptoms of anxiety and depression [41, 44]. The fact that self-esteem often decreases even more during adolescence [42], and the possibility of improving self-esteem by suitable interventions [38], makes focusing on this aspect important in interventions targeting children with internalizing symptoms.

Lastly it is worth mentioning that the current study took place in a school setting. Previous studies have pointed at the association between mental health problems and school functioning, more specifically by reducing learning capacities, increasing risk for absenteeism and academic underachievement [16]. Such problems may again influence mental health negatively. These reciprocal, negative associations are important indicators for the necessity in reaching these children with suitable and effective interventions.

Our study extends earlier research by showing that there exists a relationship between symptom levels and quality of life and self-esteem for children with depressive symptoms and for children having both depressive and anxious symptoms. This indicates the importance of always screening for depressive symptoms in preventive work and in treatment of internalizing symptoms. Assessing how such symptoms influence the child’s self-reported quality of life may give important additional information about problem severity.

We would argue that the present findings make it plausible to intervene for children who are at-risk, although not disordered, as they report lower quality of life and reduced self-esteem with increasing symptomatology. Both national [26] and international research [25, 66] have documented that children with internalizing disorders are not receiving the needed services. While many of the children reporting symptoms of anxiety and/or depression in this study would not qualify for a diagnosis, there is ample research indicating that even having fewer symptoms of anxiety and depression may render the children at risk for developing more serious problems [10]. It is also possible that some of the high-scoring children in the present sample could qualify for a disorder although this was not the focus in this study. We would therefore argue that experiencing high levels of internalizing symptoms indicate that the child could be a target for preventive efforts. The results concerning the different severity level in both the Depression only and in the Combined group on the one hand compared to the Anxiety only group on the other hand might have implications for expected change of a common indicative program, and might have implications for the emphasis given to specific interventions in such an intervention.

### Strengths and limitations

The present study has several strengths and limitations. The sample was geographically diverse and from small and large schools in both urban and rural areas. There were few missing data, and the screening measures had good psychometric properties.

While we intended a full screening of the entire target population, this was not acceptable to the ethical committee and not according to cultural norms in Norway. The sample was therefore self-selected based on the children’s own experience of being sad or anxious, and the children being screened most probably has a higher problem loading than the general child population this age.

While recruiting the children from a school setting has its advantages, some children might not be reached by the recruitment method used in this study (children with certain problems, e.g. socially anxious children, migrant children with a different cultural background).

The rating scales used are brief and cost-effective and identifies children in need of services [54] and it has been argued that self-report of internalizing difficulties can be superior to other/parent report [67]. However, inclusion of other informants of child symptoms may nevertheless add to the accurate identification of children in need. Lastly, although the cutoff scores were based on an acceptable rationale, the selection based on different means for including children to the study could have influenced the results.

## Conclusion

Schoolchildren wanted to participate in a study targeting symptomatic children with regard to anxiety and depression, and approximately half of the screened children self-reported high levels of symptoms of anxiety, depression or both. The largest at-risk group comprised of children self-reporting both depressive and anxious symptoms.

High levels of depressive symptoms and the combination of anxious and depressive symptoms were associated with reduced quality of life and self-esteem, but not symptoms of anxiety alone. A transdiagnostic approach targeting both symptom groups may be promising as a preventive or early intervention approach. Focus on enhancing self-esteem could be important in such an intervention especially so for children with depressive or mixed symptomatology. In addition, tailoring the transdiagnostic intervention might be important to get sufficient attention to children with specific challenges related to depressive or mixed symptomatology.

## Abbreviations

BSCI-Y: Beck youth inventory-II questionnaire, self-concept scale
KINDL: Kinder Lebensqualität Fragebogen questionnaire
MASC-C: Multidimensional anxiety scale for children questionnaire
SMFQ: Short mood and feelings questionnaire
